# A commentary on “Development and validation of a clinical decision tool for predicting long-term pain reduction following laparoscopic cholecystectomy in patients with symptomatic cholecystolithiasis; a prospective cohort study”

**DOI:** 10.1097/JS9.0000000000002743

**Published:** 2025-08-25

**Authors:** Hui Shang, Xianqiang Liu, Guocai Li

**Affiliations:** a Department of Anesthesiology, Shenzhen Hospital (Fu Tian) of Guangzhou University of Chinese Medicine, Shenzhen, People’s Republic of China; b Graduate School, Medical School of Chinese PLA, Beijing, People’s Republic of China

*Dear Editor,*

I have carefully read the recent article published in your journal on the development of a predictive model for long-term pain relief following laparoscopic cholecystectomy (LC)^[1]^. The authors constructed and validated a model based on a large multicenter cohort to support clinical decision-making. The study incorporated multiple clinical variables and developed an online calculator to facilitate practical use, efforts that deserve recognition. However, we would like to offer the following suggestions for the authors’ and editors’ consideration:

1. **Limitations in Predictor Selection**

While the model includes clinical variables such as sex, preoperative visual analog scale (VAS) score, pain radiation, and associated symptoms like nausea and vomiting, it appears to overlook key non-physical factors, such as psychological status and dietary habits, that may significantly influence postoperative pain outcomes. Although the authors mention psychological factors, the assessment lacks specificity regarding symptoms commonly linked to abdominal complaints, such as anxiety, depression, or perceived health status. This could potentially limit the model’s predictive accuracy.Furthermore, the study suggests that male patients, those with higher preoperative VAS scores, radiating pain, and nausea, are more likely to experience long-term pain relief. This warrants further discussion regarding the interaction between subjective pain perception and psychological modulation. Previous research has suggested, for example, that men may have higher pain tolerance thresholds and thus be more likely to report subjective improvement^[2]^; Similarly, patients with high preoperative pain levels may perceive substantial relief even if pain does not resolve completely^[3]^. These observations highlight the need for a more comprehensive approach to predictor selection, incorporating both objective clinical indicators and subjective psychological factors.

**2. External Validation and Model Applicability**

Although the model was externally validated and demonstrated a C-statistic of 0.75, indicating reasonable discriminative ability, the study did not clarify how the model performs across specific patient subgroups, such as those with functional gastrointestinal disorders (FGIDs). Given that FGID patients often experience less favorable postoperative outcomes, the model’s reliability and clinical value in such populations merit further investigation.

**3. Appropriateness of the Outcome Measure**

The primary outcome was defined as “clinically significant pain relief” rather than complete absence of pain. While this aligns with existing consensus, it may not fully reflect patient expectations, which often center on complete or near-complete pain resolution. Future research might consider incorporating multi-tiered outcome measures to provide a more nuanced prognosis.

**4. Complexity of Clinical Decision-Making**

While predictive models can support clinical decisions, real-world pain outcomes are influenced by many variables, including clinician expertise, patient expectations, and postoperative care. Although the authors acknowledge the importance of shared decision-making, overreliance on model predictions may risk overlooking individual variability and the evolving nature of patient experiences.

In conclusion, this study offers a valuable framework for predicting long-term outcomes after LC. However, improvements in predictor selection, subgroup analysis, outcome definitions, and data comprehensiveness are needed to enhance the model’s clinical utility. We hope future research will incorporate a broader range of clinical and psychosocial factors and seek validation across diverse populations, thereby improving patient management in routine practice.

## Data Availability

Not applicable.
